# Digital Natives’ Intentions Toward Informal Digital English Learning: The Roles of Desire, Engagement, and Online Learning Self-Efficacy

**DOI:** 10.3390/jintelligence14010013

**Published:** 2026-01-07

**Authors:** Fang Fang, Yaru Meng, Lingjie Tang, Yu Cui

**Affiliations:** 1 School of Foreign Studies, Xi’an Jiaotong University, Xi’an 710049, China; fangfang@sust.edu.cn (F.F.); yucui@stu.xjtu.edu.cn (Y.C.); 2 School of Culture and Education, Shaanxi University of Science and Technology, Xi’an 710021, China

**Keywords:** informal digital learning of English, digital nativity, behavioral intention, desire, engagement, online learning self-efficacy

## Abstract

Against the backdrop of rapid technological development, informal English learning has become increasingly prominent in language education, particularly among digital natives. However, limited research has examined how digital nativity influences learners’ emotions and their intentions to engage in such learning. This study investigates the relationships among digital nativity, desire, engagement, online learning self-efficacy (OLSE), and learners’ intentions toward informal digital English learning (IDLE). Data were collected from 1458 English learners and analyzed using a structural modeling approach. The results show that desire, engagement, and online learning self-efficacy play significant mediating roles in the relationship between digital nativity and learning intention, while digital nativity also exerts a direct effect on intention. These findings highlight the central role of affective and motivational factors in shaping digital natives’ learning behavior and provide empirical support for the educational value of informal digital English learning in contemporary digital environments.

## 1. Introduction

Marc Prensky coined the term “digital natives” to refer to individuals born into the digital era who demonstrate innate fluency with technology, the internet, and digital forms of communication (Prensky, 2001; X. Q. Wang et al., 2022). In the field of language learning, digital natives are particularly inclined to engage in informal approaches to learning English (Pooley et al., 2019; C. H. Wu et al., 2023) and seek English learning experiences that are engaging, adaptable, and readily available (Rezai et al., 2024). Meanwhile, a key characteristic of IDLE is the incorporation of digital technologies into learning activities conducted outside the formal classroom. Unsurprisingly, research has documented that most digital natives enjoy using digital platforms to study English beyond the formal school setting (Soyoof et al., 2023).

For language education research, IDLE has come to occupy an irreplaceable role due to its remarkable potential for enhancing language proficiency in the field of vocabulary acquisition (Lee, 2019), learner autonomy (Han & Reinhardt, 2022; Tang et al., 2024), self-regulated learning (Portus-Torres et al., 2024), willingness to communicate (Lee & Drajati, 2019) and overall language proficiency (Guan et al., 2025). Despite the documented benefits of IDLE activities for language learners, there remains a need to explore how digital nativity contributes to behavioral intention towards IDLE. Learners’ intention serves as a predictor of actual behavior (Ajzen, 1991; Lai et al., 2022; Liu & Wang, 2024). According to Ajzen (1988), intention encompasses the motivational factors driving behavior, reflecting the extent of effort that digital natives are willing to invest to engage in IDLE behavior.

Furthermore, it is increasingly acknowledged that emotional variables are central to successful language acquisition (Q. Wang et al., 2025). Researchers have explored a range of psychological factors that shape how individuals learn and use new languages, including well-being (Y. L. Wang et al., 2023), self-efficacy (Cui & Meng, 2023), resilience (Gao et al., 2025), and flow (Özhan & Kocadere, 2020). Despite these psychological factors undoubtedly playing a crucial role, desire, engagement, and OLSE remain significant yet relatively underexplored in the context of language learning. A large portion of prior research has approached these psychological factors from teachers’ perspectives (Liu & Wang, 2024), giving far less attention to how they affect learners themselves, especially within informal language-learning environments.

To fill the current research gaps, this empirical study employs a quantitative research method to investigate the mediating effects of three psychological variables—resilience, engagement, and OLSE—on the association between digital natives and IDLE intention. Our study introduces two notable innovations: First, by utilizing a substantial sample of Chinese digital natives, the study offers new empirical evidence for a group that has received limited attention in previous research. Second, this study makes a distinctive contribution by simultaneously investigating the mediating roles of desire, engagement, and OLSE within a single integrated SEM model. By bringing together emotional and cognitive variables, it sheds light on how digital nativity shapes intentions toward IDLE and generates valuable implications for instructional practice.

## 2. Literature Review

### 2.1. IDLE

IDLE comprises self-driven, interest-driven, and technology-mediated English use across a variety of digital platforms, spanning both receptive and productive activities (Lee & Dressman, 2018). It is generally defined as self-directed, voluntary English learning that occurs through engagement with digital tools and online environments outside formal instructional settings, enabling learners to acquire language authentically and autonomously” (Lee, 2019; Lee & Dressman, 2018). It occurs outside formal classroom settings, where learners use English for communication, entertainment, or self-development (Lee, 2019). Unlike structured curricula, IDLE is embedded in digital ecosystems such as social media, gaming, streaming platforms, and online communities (Sockett, 2014). Diversity of IDLE activities predicts outcomes more robustly than raw quantity of IDLE alone (Lee, 2019). Syntheses further frame IDLE as a content ecology in which authentic audience participation and focused meaning engagement matter for learning (Soyoof et al., 2023).

Empirical studies associate IDLE with gains in overall proficiency, vocabulary, and communicative readiness, especially when activities are varied and interactive rather than purely consumptive (Lee & Dressman, 2018). Contrary to the belief that simply spending more time leads to more vocabulary retention, diverse and contextualized encounters tend to produce larger gains than raw exposure (Lee, 2019). Systematic reviews converge that IDLE contributes meaningfully in EFL settings, but effect sizes depend on the activity profile and learner characteristics (X. Guo & Lee, 2023).

Across studies, affective factors such as enjoyment, motivation, self-confidence, and lower anxiety are both antecedents and outcomes of IDLE, shaping entry, persistence, and returns (Lee, 2020; Zadorozhnyy & Lee, 2023). Positive affect consistently predicts greater IDLE participation, while negative affect constrains engagement (Lee, 2020; Zadorozhnyy & Lee, 2023). Recent reviews indicate that IDLE initiates a self-reinforcing sequence in which diverse, authentic activities boost self-efficacy, increase participation, and advance skills; these effects differ by learner profile (Soyoof et al., 2023).

Digital nativity, further matching off-campus courses, captures individual differences in upbringing with technology, fluency with multitasking, preference for graphics, and desire for instant feedback (Teo, 2013). These traits correlate with technology adoption intentions to shape online English learning behaviors (Milutinović, 2022). Emerging evidence links higher digital nativity to stronger intentions to learn digital English outside formal curricula (Hui et al., 2023; M. Zhao, 2023). However, empirical work examining digital natives’ behavioral intention toward IDLE is limited, let alone the mediating effects of affective factors.

### 2.2. Digital Nativity

Digital natives are the generations raised in a digital world. They typically exhibit high proficiency with digital devices and platforms, including the internet, smartphones, and social media (Prensky, 2001). Digital nativity is a psychological construct that captures individual differences in technology use patterns, comfort with multitasking, preference for graphic communication, and a tendency toward instant gratification, as measured by the Digital Nativity Assessment Scale (DNAS; Teo, 2013). Cross-context validations further support this four-factor structure (Ursavaş et al., 2016).

Digital nativity has reshaped language-learning practices and sparked considerable scholarly attention (Cladis, 2020; Mao et al., 2014; Bittman et al., 2011). Within the English learning context, learning experiences have been reshaped by digital nativity. Digital platforms provide systematic support for the development of English-learning skills (Son, 2018), and familiarity and comfort with these platforms position learners to benefit from digital English-learning environments (Karimah & Muslim, 2019). Digital nativity also plays a role in shaping learners’ behavioral intention toward digital English learning. The effective adoption of digital tools depends not on the devices themselves, but on digital natives’ metacognitive awareness and willingness to use the devices (Rahimi & Katal, 2012). When digital devices match digital natives’ preferred ways of learning, they can help nurture favorable behavioral intention toward English acquisition (Getie, 2020). Furthermore, much research has examined the link between digital nativity and affective factors in learning, such as confidence (Šorgo et al., 2017), self-regulation (X. Q. Wang et al., 2022), motivation (M. Zhao, 2023), self-efficacy (C. Zhao & Zhao, 2021), and learning engagement (X. Q. Wang et al., 2022). For instance, C. H. Wu et al. (2023) demonstrate that self-motivation, a core component of self-regulation, is crucial for intention to learn English learning among digital natives.

Whether viewed from the digital technology perspective of digital natives or from their cognitive and emotional perspectives, these previous studies examine digital natives’ behavioral intention regarding digital English learning. Compared with classroom English learning, English learning in extramural and digital contexts is classified as IDLE (X. Guo & Lee, 2023; Zhang & Liu, 2024). However, there is limited research on IDLE intention. Hence, the following hypothesis is formulated:

**H1.**  *Digital nativity significantly predicts IDLE intention.*

### 2.3. Desire, Engagement, and OLSE

Desire is frequently regarded in psychology as a motivation laden with emotions, directed at a specific object, activity, or state, and the anticipated fulfillment is linked to pleasure (Hofmann & Van Dillen, 2012). It is closely associated with motivation (Mariqueo-Russell, 2023). In the context of English learning, desire represents an affectively charged motivation that drives learners to participate and invest in language practices (Norton, 2013). Digital natives exhibit a strong desire to learn English (Rahman Nur et al., 2023). They benefit significantly from technology-based activities, which not only foster language practice but also boost confidence. It demonstrates the intrinsic motivation of digital natives to incorporate English into their digital lives (Lamb & Arisandy, 2020). Digital natives’ active engagement with online resources, interactive learning tools, and social environments that favor English use plays a crucial role in fostering the desire for English learning outside formal classroom settings (Zeng & Zhang, 2020). Against this backdrop, we can reasonably infer that digital nativity may facilitate the development of students’ desire to engage in IDLE.

Engagement, in the context of educational research, collectively reflects a student’s level of participation, effort, interest, and investment in learning activities (Fredricks et al., 2004). For English learning, engagement refers typically to learners’ sustained cognitive, emotional, and behavioral involvement in language learning tasks, characterized by attention, persistence, and active participation, which contributes to improved learning outcomes (Philp & Duchesne, 2016). Within the language education domain, scholars have further refined this construct, with some conceptualizing engagement as encompassing cognitive, emotional, and linguistic dimensions to better align with the unique demands of language learning (Fang et al., 2025). This three-dimensional framework is adopted in the present study. Specifically within the framework of IDLE, engagement driven by autonomous use of digital tools supports autonomous English language development beyond formal classrooms (Reinders & Benson, 2017; Lee, 2019). Digital natives’ engagement is often characterized by autonomous and spontaneous interactions with digital content, suggesting a natural inclination towards informal language acquisition through digital means (Buckingham & Willett, 2006). In addition, Cakrawati (2017) reports that digital natives’ engagement with online platforms further affects their motivation and participation in English learning. Furthermore, Jurkovič (2019) suggests that high engagement towards informal digital activities contributes to perceived English competence. Against this backdrop, the engagement of digital natives with IDLE warrants further scholarly attention.

Originating from Bandura’s (1997) broader definition of self-efficacy, OLSE describes how confident people feel about their ability to carry out and succeed in online learning activities, emphasizing learners’ confidence in using digital tools, staying motivated and overcoming challenges unique to online contexts (Zimmerman & Kulikowich, 2016; Shen et al., 2013). In the digital era, self-efficacy among digital natives not only impacts their engagement with digital learning tools and resources (Lestari, 2020), but also shapes their digital competence and positive views of online language learning experiences (Katsarou, 2021), both of which are essential for effective online language education. Against this backdrop, prior research confirms that self-efficacy is a pivotal factor in digital natives’ online English learning, as it influences their confidence, engagement, competence, and overall satisfaction with digital language learning environments (Ke, 2011; Q. Wang et al., 2025). Leveraging the above views, we put forward the following hypotheses to explore the influence of digital nativity on desire, engagement, and OLSE:

**H2.**  *Digital nativity significantly predicts desire.*

**H3.**  *Digital nativity significantly predicts engagement.*

**H4.**  *Digital nativity significantly predicts OLSE.*

### 2.4. Behavioral Intention Through Desire, Engagement, and OLSE

Behavioral intention represents the strength of an individual’s intention to engage in a behavior, shaped by attitudes, subjective norms, and perceived behavioral control (Ajzen, 1988). It holds a core, indispensable position, and it is universally regarded as the most direct psychological precursor to actual behavior (M. Zhao, 2023). The more intense a person’s intention to engage in a behavior, the more likely they will execute that behavior (Ajzen, 1991). Previous studies have devoted substantial research attention to elucidating the determinants of behavioral intention across different learning contexts. In digital learning contexts, researchers have increasingly focused on the role of digital nativity in fostering learners’ behavioral intentions, such as the impact of e-learning system design and management (Bag et al., 2022) and perceived interactivity, prominence, and congruency in game-based learning (Wouters et al., 2013). In the digitized learning context, digital nativity’s affective factors are apparently pivotal, including learning motivation (Huang, 2024), enjoyment (Y. Guo et al., 2017), attitude (Faham & Asghari, 2019) and satisfaction (Ngo et al., 2025). For example, perceived ease of use and perceived usefulness are key determinants of learners’ behavioral intention to engage with learning platforms, including mobile learning environments (Chao, 2019), and virtual reality systems (Quaid et al., 2020). However, there is scarce research concerning desire, engagement, and OLSE.

As previously mentioned, desire emerges as a complex, fluid facet of the psyche, deeply intertwined with identity formation and serving as a core driver of the learning process (Deleuze & Guattari, 1987). Desire is central to motivations for learning English (Motha & Lin, 2014), and plays the pivotal role as a precursor to behavioral intention (Song et al., 2012). Meanwhile, through a mediating role, desire shaped by emotional and cognitive factors influences behavioral intention (Choi et al., 2020). Mariqueo-Russell (2023) identified that desire, along with pleasure and satisfaction, plays a key mediating role in the formation of behavioral intention. Specialized to the language learning context, desire, conceptualized as a form of intrinsic motivation, directly and indirectly impacts learners’ behavioral intention to continue language acquisition (MacIntyre & Blackie, 2012), and to engage with technology in the language learning field (Hsieh et al., 2017). Building on the above research, the psychological factor, desire, plays a pivotal role in shaping students’ behavioral intention toward IDLE within the context of English learning.

In the field of education, engagement refers to students’ active involvement in the learning process, with an emphasis on meaningful participation that fosters deep learning (Stefani, 2008), encompassing students’ behavioral, emotional, and cognitive engagement (Abdullah et al., 2015). However, researchers have found that in IDLE settings, engagement in language learning is often linked to active participation and the effective use of linguistic resources in social, contextualized situations (Arndt, 2023). Meanwhile, in informal digital learning, engagement extends to interaction with technological tools and digital resources (Henrie et al., 2015). Considering the factors mentioned above, the main dimensions of engagement in this study are defined as follows: affective, cognitive and linguistic engagement. As a key precursor to behavioral intention, engagement plays an indispensable role. Researchers have found that behavioral intention is supported by diverse engagement forms, such as, engagement with digital tools and resources (Hayadi & Hariguna, 2025), emotional and motivational involvement (Lee & Drajati, 2019), and diverse activity participation (Lee, 2019). This study further investigates the three dimensions of engagement, with a particular focus on the linguistic dimension, in relation to the intention to IDLE.

Based on social cognitive theory, self-efficacy is defined by Bandura (1997) as learners’ belief in their capability to perform specific tasks, which shapes their behavioral choices, resilience in overcoming obstacles, and ultimate performance outcomes, as widely utilized in e-learning research. In digital learning environments, OLSE consistently enhances behavioral intention both directly, by facilitating the integration and continued application of digital tools (C. Zhao & Zhao, 2021), and indirectly, through the influence of key belief constructs. For example, OLSE increases perceived ease of use and perceived usefulness, thereby elevating intention and satisfaction (Joo et al., 2011). In language learning, OLSE closely related technological self-efficacy, improves performance expectancies and attitudes, thereby boosting behavioral intention and continuance intention (Y. L. Wang et al., 2023) Though few studies explicitly model behavioral intention toward IDLE, emerging research in EFL links OLSE to IDLE participation, engagement, and satisfaction, which directly shape sustained intention. For example, OLSE positively predicts IDLE and online English course satisfaction (Zheng & Xiao, 2023); OLSE and behavioral intention act as mediators between IDLE activities and learners’ involvement (Fang et al., 2025), with stronger OLSE boosting IDLE intention. Collectively, they provide evidence for a plausible pathway from OLSE to behavioral intention in IDLE. Drawing on prior studies, OLSE tools typically focus on formal digital learning, while this study aligns OLSE items with IDLE-specific tasks. Drawing on the above perspectives, the following hypotheses are put forward to explore further the effects of desire, engagement and OLSE on behavioral intention towards IDLE:

**H5.**  *Desire significantly predicts behavioral intentions.*

**H6.**  *Engagement significantly predicts behavioral intention.*

**H7.**  *OLSE significantly predicts behavioral intention.*

### 2.5. The Hypothesized Structural Model

**Figure 1:**
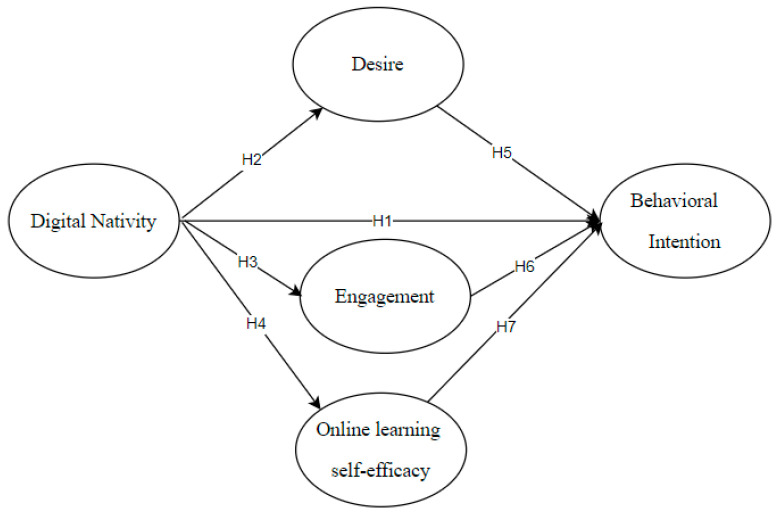
Hypothesized research model.

Figure 1 shows the proposed structural model, exploring the connections between digital nativity, mediators (desire, engagement, and OLSE), and behavioral intention toward IDLE. Hypothesis H1 stands for the predictive impact of digital nativity on behavioral intention, explaining how digital nativity supports learners’ behavioral intention for IDLE activities. Hypotheses H2 to H4 represent the predictive effects of digital nativity on desire, engagement, and OLSE, illustrating how digital nativity aids learners’ behavioral intention for IDLE activities. Hypotheses H5 to H7 test how desire, engagement, and OLSE influence behavioral intention toward IDLE, highlighting their roles in promoting intention in informal digital English learning. With a unified framework, this model facilitates the investigation of the mediating roles that desire, engagement, and OLSE play in linking digital nativity to IDLE intention.

### 2.6. Research Questions

Based on previous scholarly works, our study intends to investigate the following questions:

RQ1: Does desire mediate between digital nativity and behavioral intention toward IDLE, and in what specific ways is this role manifested?
RQ2: Does engagement mediate between digital nativity and behavioral intention toward IDLE, and in what specific ways is this role manifested?
RQ3: Does OLSE mediate between digital nativity and behavioral intention toward IDLE, and in what specific ways is this role manifested?

## 3. Methodology

### 3.1. Participants

For this study, 1458 Chinese university students were recruited through convenience sampling via the online survey platform Wenjuanxing. Participants were included if they were currently enrolled university students with prior experience in digital English learning activities. Given that contemporary university students in China are predominantly post-1990s individuals, classified as digital natives according to Prensky’s (2001) definition, they actively engage in online English practices. They are therefore well-suited for examining behavioral intentions toward IDLE. Prior to giving informed consent, every participant was thoroughly briefed on the study’s purposes, procedures, and potential ramifications, ensuring their participation was voluntary and that they fully comprehended their contributions.

Of all participants, 45.06% (*n* = 657) were male and 54.94% (*n* = 801) were female. Meanwhile, 99.2% are aged 16 to 30, and the other 12 are under 35. Among these 1458 participants, 94.86% (*n* = 1383) either hold or are pursuing an undergraduate degree, 4.87% (*n* = 71) a master’s degree, and 0.27% (*n* = 4) a doctoral degree. A closer look shows the study sample included learners majoring in English (3.36%, *n* = 49), Humanities (2.88%, *n* = 42), Social Science (16.05%, *n* = 234), Natural Science (19.20%, *n* = 280), Engineering (54.73%, *n* = 798), and other fields (3.77%, *n* = 55). It is also worth noting that all participants have experience with informal digital language learning. When it comes to the specific informal digital technologies they use most often, most opted for music (63.10%, *n* = 920) and films (64.81%, *n* = 945) in their IDLE activities, while 48.35% (*n* = 705) used books. In addition, 37.45% (*n* = 546) chose TV programs (e.g., series) and 39.23% (*n* = 572) chose social media (e.g., WeChat, Bilibili), whereas only 25.03% (*n* = 365) chose digital games.

### 3.2. Research Instrument

A revised survey tool was employed to collect the data. Modifications were made to better capture digital natives’ behavioral intentions toward IDLE. Specifically, the term “English learning activities” was adapted to “IDLE activities” and references to “the mobile learning platform” were clarified by providing examples such as “WeChat, Weibo, Xiaohongshu, Facebook, and Instagram”. This survey was split into two parts. Part one concentrated on participants’ demographic details, designed to gather information especially about their academic discipline, and technology usage experience. The second part included 34 items from five different mature scales. These scales measured digital nativity, engagement, desire, online learning self-efficacy, and behavioral intention, respectively. All participants needed to respond to these questions using a 5-point Likert scale.

#### 3.2.1. Digital Nativity Assessment Scale (DNAS)

To assess students’ digital native status, we used Teo’s (2013) questionnaire. The study checked the content validity. This scale features 14 items across four sub-constructs: reliance on graphics for communication (RG, 3 items), growing up with technology (GUT, 3 items), thriving on instant gratification and rewards (TD, 4 items), and being comfortable with multitasking (CM, 3 items). A sample item is ‘I expect quick access to information when I need it.’ Cronbach’s alpha reliability coefficients demonstrated high reliability for the overall DNAS (α = 0.904).

#### 3.2.2. Behavioral Intention Scale

Behavioral intention in the scale of this study refers more to digital natives’ intention to adopt and utilize a particular technology tool. To assess learners’ behavioral intention regarding social media use in IDLE, we made some modifications to the questionnaire from Fang et al. (2025). After the researchers’ discussion, we adopted the four items closely related to the present study. A sample item follows: ‘I intend to use the learning contents in electronic devices to enhance my learning.’ Reliability testing using Cronbach’s alpha indicated that the scale achieved high internal consistency (α = 0.947).

#### 3.2.3. Desire to Learn English Scale

To assess students’ motivation to learn English, we adapted the questionnaire from Yashima et al. (2004). Following multiple rounds of discussions with another researcher and two doctoral students, one item was removed. Moreover, during the tests of discriminant validity, the value of another item failed to meet the required criteria; thus, it was excluded, and ultimately, three items were retained in the final analysis. A sample item is ‘I strongly believe that English should be an integral part of the school curriculum.’ In the current study, Cronbach’s alpha reliability coefficient computations indicated that the overall scale had high reliability (α = 0.760).

#### 3.2.4. Engagement in IDLE Scale

To measure engagement among digital natives, we adopted a questionnaire (Arndt, 2023) originally designed to assess involvement in the context of informal second language learning (ISLE). This scale covers multiple facets of learners’ engagement with ISLE-related activities. Following several rounds of discussion among the researchers, the scale was refined to better align with the context of this study, which focuses on IDLE. The final questionnaire measuring engagement included 8 items, split into three aspects: affective, cognitive, and linguistic engagement. One item was: ‘I was completely focused on trying to understand every single word while using electronic devices.’ We calculated Cronbach’s alpha reliability coefficients, and the results indicated that the total engagement scale has high reliability (α = 0.926).

#### 3.2.5. Online Learning Self-Efficacy Scale (OLSE)

Online learning self-efficacy items were adapted from Hong et al. (2017). This construct captures learners’ confidence and perceived competence in leveraging internet-based tools for English learning. Following multiple rounds of expert evaluation, three items were kept for analysis. An example item is: “If I encounter a new challenge while using a website to learn, I can always find a strategy to overcome it.” Reliability testing indicated strong internal consistency, with the scale yielding a Cronbach’s alpha (α = 0.908).

### 3.3. Data Analysis

The analytical process was structured into four sequential steps. First, data preparation was conducted, including the removal of invalid responses (e.g., incomplete entries or patterned responses) and the identification of multivariate outliers using Mahalanobis distance. Skewness and kurtosis were assessed to evaluate univariate normality, and missing data were handled using multiple imputation to improve dataset completeness. Second, the study undertook a comprehensive evaluation of the scale’s reliability and validity. The scale’s internal consistency was measured through Cronbach’s alpha, and Confirmatory Factor Analysis (CFA) was utilized to examine the soundness of the measurement model. Convergent and discriminant validity were examined by analyzing the Composite Reliability (CR), the Average Variance Extracted (AVE), and the square root of the AVE. Third, descriptive statistics were conducted to capture the overall patterns and variability of the data. Finally, the proposed theoretical framework was evaluated through a structural model. By examining both direct and mediating paths, the study provided a comprehensive interpretation of the relationships among the key constructs.

## 4. Findings

### 4.1. Descriptive Statistics

All the items, as shown in Figure 1, meet the model criteria, with the absolute values of skewness and kurtosis less than 2 and 10, respectively. This finding confirms the dataset’s normality, in line with Collier’s (2020) criteria. Additionally, the means and standard deviations of the observed variables, can reflect the central tendency and dispersion of the latent variables. As shown in Table 1, the SD values for all items range from 0.657 to 0.846, indicating moderate variability in participants’ responses. Mean scores for digital native (and its subscales) as well as behavioral intentions exceeded 3.50, suggesting that participants held generally positive behavioral intentions toward IDLE. A detailed analysis of emotional variables reveals that engagement in IDLE and OLSE fell within the high-medium range, whereas desire was slightly below the medium level.

### 4.2. Assessment of Reliability and Validity

Reliability and validity checks were performed to verify that the research data were adequate for subsequent advanced statistical analysis. The five scales’ Cronbach’s α coefficients were 0.904 (Digital Native), 0.947 (Behavioral Intention), 0.926 (Engagement), 0.848 (Desire), and 0.908 (OLSE). As recommended by Kline (2023), all five values surpass the 0.7 benchmark, reflecting acceptable internal reliability. To test the scale’s validity, our study measured the standardized factor loadings of each item in the scale. We also checked CR and AVE for each variable. As shown in Table 2, all standardized factor loadings exceeded the threshold value of 0.5, and both CR and AVE were above the recommended cut-off values of 0.7 and 0.5 (Kline, 2023), respectively. These results provide strong evidence for the scale’s convergent validity.

To evaluate discriminant validity, we computed the Heterotrait–Monotrait Ratio of Correlations (HTMT) for each factor, following Henseler et al.’s (2015) recommendations. All HTMT values were under the level of 0.90, except for the one related to behavioral intention (0.905), which surpassed this threshold by a slight amount. Despite this minor deviation, the overall pattern indicates that the measure achieved satisfactory discriminant validity.

As shown in Table 3, AMOS was used to construct the measurement model and evaluate its structural validity. Model fit was assessed using six key fit indices. The results indicated that all indices met the recommended criteria, demonstrating a good model fit. Specifically, the values were as follows: χ^2^/df = 3.584, Incremental Fit Index (IFI) = 0.985, Tucker–Lewis Index (TLI) = 0.980, Comparative Fit Index (CFI) = 0.985, Root Mean Square Error of Approximation (RMSEA) = 0.042, and Standardized Root Mean Square Residual (SRMR) = 0.027.

### 4.3. Structural Model Evaluation and Hypothesis Testing

After establishing an acceptable fit for the measurement model, this study proceeded to evaluate the structural model. According to the recommended fit index criteria, the structural model demonstrated good fit. The specific values were as follows: χ^2^/df = 3.662, CFI = 0.984, IFI = 0.984, TLI = 0.979, SRMR = 0.026, RMSEA = 0.043, with a 90% confidence interval of [0.037, 0.047], and a PCLOSE value of 0.997. As shown in Table 4, the mediation model’s path coefficients fully supported all seven hypotheses. The findings indicate that digital nativity exerts significant positive predictive effects on desire, engagement, OLSE, and behavioral intention. The specific path coefficients were as follows: desire (β = 0.345, *p* < 0.001, t = 10.881), engagement (β = 0.604, *p* < 0.001, t = 19.055), OLSE (β = 0.515, *p* < 0.001, t = 17.361), and behavioral intention (β = 0.151, *p* < 0.001, t = 5.355). Further analysis revealed that desire, engagement, and OLSE were all significant predictors of informal English learning. Their path coefficients were: desire (β = 0.265, *p* < 0.001, t = 8.020), engagement (β = 0.222, *p* < 0.001, t = 5.913), and OLSE (β = 0.277, *p* < 0.001, t = 8.397).

As presented in Table 5, it is crucial to emphasize that the paths “Digital Nativity → Desire → Behavioral Intention,” “Digital Nativity → Engagement → Behavioral Intention,” and “Digital Nativity → OLSE → Behavioral Intention” were all significantly related, which means that the basic assumption of the mediation test is fully satisfied. Additionally, we implemented a mediating analysis approach to explore the link between digital nativity and behavioral intention. For this analysis in AMOS, a bootstrapped approach with 5000 samples and confidence intervals at the 95% level was utilized. The findings indicated that the indirect effect of “Digital Nativity → Desire → Behavioral Intention” stood at 0.124, with a lower bound of 0.084 and an upper bound of 0.170. The mediating effect of “Digital Nativity → Engagement → Behavioral Intention” stood at 0.182, with a lower bound of 0.115 and an upper bound of 0.257. For “Digital Nativity → OLSE → Behavioral Intention,” the indirect effect stood at 0.193, with a lower bound of 0.132 and an upper bound of 0.262. Because zero did not fall within either confidence interval, the mediating relationships were significant (*p* < 0.01). To put it another way, digital nativity can shape behavioral intention indirectly through the partial mediation of desire, engagement, and OLSE.

**Figure 2:**
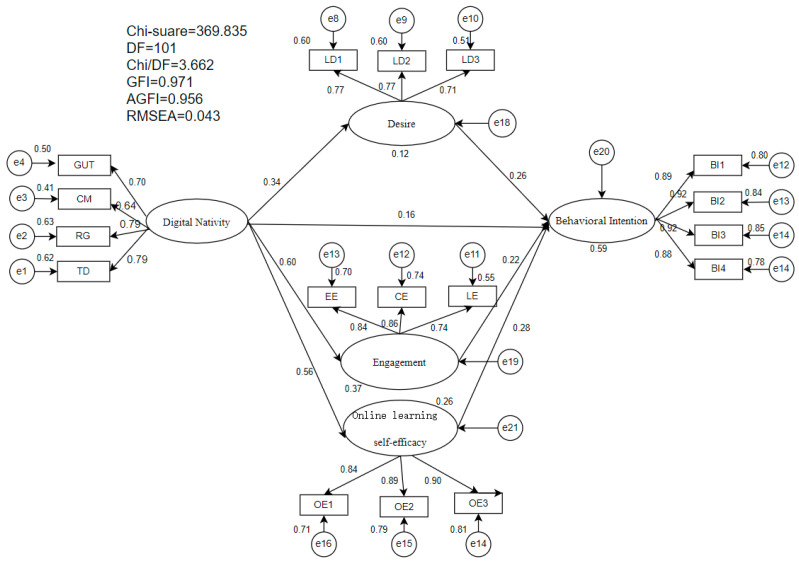
The final structural model. Note: (1) GUT = grow up with technology; CM = comfortable with multitasking; RG = reliant on graphics for communication; TD = thrive on instant gratification and rewards; LD = desire; BI = behavioral intention; EE = affective engagement; CE = cognitive engagement; LE = linguistic engagement. (2) R^2^ (Desire = 12%; Engagement = 37%; Online learning self-efficacy = 27%; Behavioral Intention = 59%).

As shown in Figure 2, the R^2^ values indicated that Digital Nativity explained 12%, 37%, and 27% of the total variance in desire, engagement, and OLSE, respectively. Meanwhile, the combined impacts of the three factors accounted for a notable 59% of the variance in behavioral intention, indicating that the model explained 59% of the variation in this construct. These results support the model’s explanatory power when it comes to understanding EFL digital natives’ behavioral intention toward IDLE.

## 5. Discussion

### 5.1. Desire in IDLE: Motivational Amplifier and Commitment Reinforcer

Regarding the mediating role of desire in Research Question 1, we offer the following discussion. Our findings indicate that desire plays a crucial mediating role between digital nativity and behavioral intention toward IDLE. This finding posited that digital natives with strong intrinsic motivation for language learning tend to have intention towards IDLE activities, thereby strengthening their willingness to pursue such learning behaviors consistently. The mediating role of desire suggests that when digital natives perceive IDLE as a means to fulfill their personal language-related goals, their desire for learning is activated and amplified (Lee & Drajati, 2019). This activated desire then exerts a positive influence on their behavioral intention towards IDLE, as it bridges the gap between their learning needs and the perceived value of informal digital learning contexts (Ryan & Deci, 2017). Compared with previous studies, which have largely focused on the direct effects of digital literacy on IDLE learners (Gao & Li, 2019), our research extends these findings by identifying desire as a critical intermediary factor, highlighting that even high digital literacy may not translate into strong behavioral intention without a corresponding desire to engage in IDLE. The significance of this mediating role highlights the necessity of learning strategy practices that prioritize nurturing digital natives’ intrinsic desire for IDLE, ultimately leading to stronger behavioral intention and finally effective IDLE.

In addition, our study underscores that desire significantly mediates behavioral intention towards IDLE among digital natives. First, desire strengthens the link between perceived content value and learning intention. Digital natives, who see educational, entertainment, or social content in IDLE as valuable, develop a strong desire, which turns abstract value judgments into concrete behavioral intention, as learners actively seek needs-aligned content. This aligns with Bhatti and Aldubaikhi (2023), who found that diverse digital content correlates with greater personal desire and subsequent motivation for IDLE. Second, desire narrows the gap between intrinsic motivation and sustained action. Digital natives often have an inherent interest in tech-integrated learning, but this alone rarely leads to consistent IDLE participation. Desire acts as a motivational amplifier, sustaining attention during challenges and reinforcing long-term commitment to practice. This supports self-determination theory, which notes that desire rooted in autonomy and relevance mediates between intrinsic motivation and goal-directed behavior (Ryan & Deci, 2017). Third, desire facilitates adaptation to IDLE’s dynamic environments. Unlike formal education, IDLE lacks structured guidance, demanding learner self-direction. Digital natives with strong English proficiency are more willing to test new platforms, adjust strategies, and persist through obstacles, which boosts their adaptability and intention to keep engaging in IDLE. This aligns with Venkatesh and Davis’s (2000) identification of desire as a key mediator between perceived technology utility and sustained usage intention.

### 5.2. Engagement in IDLE: Anxiety Reducer and Efficiency Promoter

Concerning the mediating role of engagement in Research Question 2, we elaborate on our discussions as follows: Our study finds that engagement significantly mediates the link between digital nativity and IDLE behavioral intention, aligning with Zhang and Liu (2024), who showed that digital natives’ inherent digital familiarity drives deeper informal learning engagement, which boosts sustained behavioral intention. Engagement bridges the gap between digital nativity and consistent IDLE participation by channeling digital proficiency into purposeful interactions with learning content. This aligns with the transactional model of engagement, which posits that meaningful engagement acts as a critical mediator between digital competencies and goal-directed learning behaviors. Compared to prior research focusing on formal online learning, our study emphasizes engagement’s unique role in IDLE environments, where unstructured resources and diverse platforms demand continuous active involvement. This mediating effect echoes findings by R. L. Wu (2023), noting that engagement, shaped by digital learners’ adaptive skills, serves as a key link between informal learning participation and sustained behavioral commitment. Practically, by designing IDLE platforms to prioritize interactive features that foster engagement, educators can leverage digital natives’ strengths to boost learning intentions, enhancing individual consistency and enriching the broader IDLE ecosystem.

Our study further emphasizes that engagement strongly mediates digital natives’ behavioral intention toward IDLE through three key pathways: firstly, it boosts their English learning commitment; digital learners who perceive themselves as effective in their learning roles tend to be more engaged, which promotes more profound commitment to digital English learning, brings a feeling of fulfillment, and thereby fosters sustained IDLE intention, aligning with Capone and Lepore (2022), who highlighted that maintaining learner engagement is central to sustaining motivation and participation in digital learning in remote contexts. Secondly, higher digital English engagement reduces technology-related stress. Digitally native learners actively engaged in IDLE roles feel less overwhelmed by platform demands or technical complexities, which lowers stress and strengthens their intention to continue, echoing Klassen and Chiu (2010), who found that meaningful engagement with digital tools mitigates technology-related anxiety. And thirdly, it promotes English proficiency; engaged IDLE learners are more likely to engage deeply with language content, forming a cycle of skill development and positive experiences, and this proficiency growth further enhances their intention to sustain IDLE participation.

### 5.3. Online Learning Self-Efficacy in IDLE: Emotion Regulator and Technology Adapter

Regarding the mediating role of OLSE addressed in Research Question 3, we offer the following discussion. Our study finds that OLSE significantly mediates the relationship between digital nativity and behavioral intention toward IDLE. This aligns with the findings of X. Q. Wang et al. (2022), who noted that digital learners with self-efficacy are better able to cope with the helplessness and frustration encountered during the learning process. Self-efficacy helps English learners maintain a positive attitude and confidence when facing difficulties, which, in turn, reinforces their intention to engage in digital language learning (Zheng & Xiao, 2023). The mediating function suggests that digital nativity not only can elevate the confidence levels of English learners but also cultivates self-efficacy, enabling them to tackle challenges more effectively (C. Zhao & Zhao, 2021). Unlike previous research that focused on formal English learning, our study shows that OLSE plays a vital role in IDLE, as learners may feel frustrated or helpless when faced with various technical issues or the immediate feedback generated by digital platforms. The broader implications of this finding are significant for learning policies and practices. The strategies aimed at enhancing OLSE in the design of informal English learning platforms and related programs, and at offering continuous support, help learners develop the OLSE required to sustain their confidence and intentions toward IDLE. This approach may result in more stable and satisfied English learners, ultimately benefiting the wider language-learning community.

Additionally, our study emphasizes that OLSE serves as a significant mediator of digital natives’ behavioral intention toward IDLE, which can be explained from three key perspectives. First, OLSE plays a vital role in managing helplessness. Learners with strong digital self-efficacy trust their ability to tackle online English-learning challenges, which strengthens their OLSE. With this OLSE, digital natives can effectively handle frustration during learning, reduce burnout, and ultimately boost their overall intention to engage in IDLE. This result aligns with S. Wu and Tu (2019), who noted that learners with robust self-efficacy are better able to cope with learning helplessness and maintain their participation intentions in digital language contexts. Second, OLSE facilitates positive adaptation to technical difficulties. Learners with high digital nativity feel confident in overcoming technical obstacles, which reinforces their OLSE. Positive adoption can help alleviate feelings of helplessness, enhance a sense of achievement, and, in turn, improve their intentions toward IDLE. This observation supports the foundings of R. L. Wu (2023), who found that self-efficacy is critical for English learners to adapt to the changing demands of informal digital learning environments. Third, OLSE supports emotional regulation, helping English learners maintain stable emotions. Learners with a high level of digital literacy can mobilize their own OLSE to regulate their responses to stress. Effective emotional management can reduce negative feelings and enhance positive emotions, thereby effectively boosting the intention to engage in IDLE. This is consistent with R. L. Wu’s (2023) findings, which noted that English learners with high self-efficacy are better at managing emotions during digital learning—a skill essential for sustaining long-term participation intentions.

### 5.4. Theoretical and Practical Implications

This study makes a substantial contribution to SDT from a theoretical standpoint. By focusing on the IDLE activities of Chinese digital natives, it expands the theory’s application field and highlights the relevance of the SDT constructs across multiple areas of education. Additionally, the study identifies desire, engagement, and OLSE as mediating factors in the relationship between digital natives and their behavioral intentions toward IDLE, offering a detailed understanding of the underlying mechanisms. This study incorporates the cognitive and psychological factors of desire, engagement, and OLSE into the SDT framework within the field of education. This framework offers a theoretical basis for developing intervention strategies that can effectively enhance behavioral intention toward IDLE and improve the efficiency of English learning.

In addition, the study provides several practical implications for improving English pedagogical strategies in universities. Given the widespread engagement of digital natives in IDLE and the significant role of emotion regulation, educators should integrate IDLE into instructional design while simultaneously embedding emotion regulation strategies into the curriculum. Firstly, to effectively incorporate IDLE into instructional design, support systems should be established within universities to organize IDLE workshops and technical support hubs. Meanwhile, teachers can guide students in selecting suitable tools based on proficiency and host in-class sessions to share IDLE progress (Reinders & Benson, 2017; Soyoof et al., 2023; Q. Wang & Pan, 2023). This helps digital natives navigate unstructured IDLE, link informal learning to formal goals, reduce technical anxiety, and build a practice community, holistically creating a scaffolded IDLE ecosystem that boosts engagement, proficiency, and overall English learning success. Secondly, this study offers insights into emotion regulation strategies by highlighting the roles of desire, engagement, and OLSE. Desire acts as an intrinsic motivational amplifier, reinforcing long-term commitment to practice. Teachers should incorporate needs-aligned digital content, providing ongoing support to help learners build inherent motivation to tech-integrated learning, adapt to challenges, and maintain an optimistic attitude. Engagement boosts English learning commitment. Digital teaching platforms should add real-time support to provide progress feedback, fostering purpose and sustaining learners’ intentions. Furthermore, digital engagement can reduce tech-related stress, thereby strengthening their intent to continue IDLE. To enhance students’ OLSE, curriculum design should incorporate more course tasks that involve online digital technologies, all of which reinforce their long-term IDLE intentions.

## 6. Conclusions

This study conducts an investigation into the mediating roles of desire, engagement, and OLSE in the correlations between digital nativity and behavioral intention towards IDLE by means of SEM. Our results offer functional understandings of how digital natives perceive IDLE and clarify the factors that impact behavioral intention. The outcomes showed that digital natives with stronger nativity levels usually had positive behavioral intention towards IDLE. Our findings also uncovered that desire, engagement, and OLSE act as complete mediators in the relationship between digital nativity and IDLE intention. Desire emerges as a critical mediator of IDLE intention, connecting perceived content value to action, closing gaps between intrinsic motivation and sustained behavior, and supporting adaptation to dynamic IDLE settings. These roles underscore desire’s comprehensive impact on strengthening digital natives’ IDLE behavioral intention by effectively linking learning needs, value, and ongoing involvement. Engagement emerges as a pivotal mediator of IDLE intention, enhancing learning commitment, alleviating tech-related stress, and fostering English proficiency. These roles underscore engagement’s comprehensive impact on sustaining digital natives’ IDLE behavioral intention by bridging digital nativity and successful navigation of unstructured IDLE environments. OLSE emerges as a key mediator of IDLE intention, addressing learning helplessness, supporting technical adaptation, and enabling emotional regulation, which shows OLSE’s comprehensive impact on promoting digital natives’ IDLE behavioral intention by facilitating effective navigation of IDLE’s unique challenges. Together, the theoretical contributions to SDT and the practical implications of desire, engagement, and OLSE illuminate the mechanisms that shape digital natives’ IDLE intentions. These research findings provide valuable references for teachers and policymakers seeking to promote the integration of IDLE into university English teaching and to improve English learning.

This study has several limitations to acknowledge. First, the quantitative data of this study are derived solely from questionnaires. This single-collection method has limitations for understanding the participants’ comprehensive situation, thereby reducing the persuasiveness of the data. Second, a limitation of this study is the omission of qualitative methodologies, including interviews, focus groups, and narrative-based inquiry. Qualitative research can effectively assist in interpreting quantitative data and in better explaining the roles of desire, engagement, and OLSE among digital natives in IDLE. To better address these limitations, future research would benefit from integrating both quantitative and qualitative methodologies. Additionally, it would be meaningful to investigate the influence of IDLE contexts on the dynamic changes in affective and cognitive factors, such as desire, engagement, and OLSE. A longitudinal study is a highly effective research method for analyzing the long-term developmental laws of constructs. It can clearly identify the key developmental periods and the most critical external influencing factors which affect IDLE intention over time. These improvements would deepen understanding of the mechanisms driving digital natives’ IDLE behavioral intention, laying the groundwork for more targeted and effective educational interventions.

## Figures and Tables

**Table 1:** Descriptive statistics and Factor loading (In CFA).

Factors		M	SD	Kurtosis	Skewness	Factor Loading	α (>0.7)
Digital Nativity	GUT	3.81	0.827	0.309	−0.512	0.758	0.904
	CM	3.52	0.803	0.106	−0.240	0.657	
	RG	3.78	0.736	0.331	−0.304	0.769	
	TD	3.99	0.657	0.664	−0.432	0.751	
Behavioral Intention	BI1	3.66	0.777	0.479	−0.341	0.893	0.947
	BI2	3.67	0.769	0.574	−0.426	0.919	
	BI3	3.68	0.763	0.508	−0.352	0.924	
	BI4	3.66	0.787	0.586	−0.399	0.883	
Engagement	EE	3.82	0.700	0.631	−0.252	0.828	0.926
	CE	3.58	0.671	0.494	−0.030	0.865	
	LE	3.53	0.668	0.705	−0.093	0.832	
Desire	LD1	3.24	0.793	0.330	0.035	0.707	0.848
	LD2	3.72	0.844	0.571	−0.558	0.718	
	LD3	3.27	0.846	0.391	−0.192	0.736	
Online learning self-efficacy	OLSE1	3.51	0.734	0.104	0.033	0.868	0.908
	OLSE2	3.51	0.750	0.193	−0.057	0.913	
	OLSE3	3.53	0.738	0.206	−0.041	0.882	

Note: OLSE = online learning self-efficacy; GUT = grow up with technology; CM = comfortable with multitasking; RG = reliant on graphics for communication; TD = thrive on instant gratification and rewards; LD = desire; BI = behavioral intention; EE = affective engagement; CE = cognitive engagement; LE = linguistic engagement.

**Table 2:** Convergent Validity and Discriminant Validity.

		AVE (>0.5)	CR (>0.7)	HTMT (<0.9)				
				Digital Native	Behavioral Intention	Engagement	Desire	OLSE
1	Digital Nativity	0.540	0.824	0.735				
2	Behavioral Intention	0.819	0.948	0.555	0.905			
3	Engagement	0.708	0.879	0.603	0.691	0.841		
4	Desire	0.519	0.764	0.384	0.665	0.671	0.720	
5	OLSE	0.789	0.918	0.519	0.681	0.718	0.653	0.888

Note: OLSE = online learning self-efficacy.

**Table 3:** Model Fit Indices.

	χ^2^/df	CFI	IFI	TLI	RSMEA	SRMR
The measurement model	3.584	0.985	0.985	0.980	0.042	0.027
The structural model	3.662	0.984	0.984	0.979	0.043	0.026
Cutoff values (Kline, 2023)	<5	>0.90	>0.90	>0.90	<0.10	<0.08

**Table 4:** Hypothesis test results.

Path	β	*p*	t-Value	Results
Digital Nativity → Behavioral Intention	0.151	***	5.355	accepted
Digital Nativity → Desire	0.345	***	10.881	accepted
Digital Nativity → Engagement	0.604	***	19.055	accepted
Digital Nativity → OLSE	0.515	***	17.367	accepted
Desire → Behavioral Intention	0.265	***	8.020	accepted
Engagement → Behavioral Intention	0.222	***	5.913	accepted
OLSE → Behavioral Intention	0.277	***	8.397	accepted

Note: (1) *** *p* < 0.001; (2) OLSE = online learning self-efficacy.

**Table 5:** Goodness-of-fit indices of the measurement models.

Mediation Paths	95% Confidence Interval		*p* (Two-Tailed Significance)	Indirect Effect	Results
	Lower Bound	Upper Bound			
Digital Nativity → Desire → Behavioral Intention	0.084	0.170	0.000	0.124	accepted
Digital Nativity → Engagement → Behavioral Intention	0.115	0.257	0.000	0.182	accepted
Digital Nativity → OLSE → Behavioral Intention	0.132	0.262	0.000	0.193	accepted

Note: OLSE = online learning self-efficacy.

## Data Availability

The data presented in this study can be made available upon reasonable request from the corresponding author.
